# Geriatric Helper: An mHealth Application to Support Comprehensive Geriatric Assessment

**DOI:** 10.3390/s18041285

**Published:** 2018-04-22

**Authors:** Samuel Silva, Rafael Felgueiras, Ilídio C. Oliveira

**Affiliations:** 1 Department of Electronics, Telecommunications and Informatics, University of Aveiro, 3810-193 Aveiro, Portugal; rafaelfelgueiras@ua.pt (R.F.); ico@ua.pt (I.C.O.); 2 Institute of Electronics and Informatics Engineering of Aveiro, University of Aveiro, 3810-193 Aveiro, Portugal

**Keywords:** mHealth, comprehensive geriatric assessment, user centred design

## Abstract

The Comprehensive Geriatric Assessment (CGA) is a multidisciplinary diagnosis approach that considers several dimensions of fragility in older adults to develop an individualized plan to improve their overall health. Despite the evidence of its positive impact, CGA is still applied by a reduced number of professionals in geriatric care in many countries, mostly using a paper-based approach. In this context, we collaborate with clinicians to bring CGA to the attention of more healthcare professionals and to enable its easier application in clinical settings by proposing a mobile application, Geriatric Helper, to act as a pocket guide that is easy to update remotely with up-to-date information, and that acts as a tool for conducting CGA. This approach reduces the time spent on retrieving the scales documentation, the overhead of calculating the results, and works as a source of information for non-specialists. Geriatric Helper is a tool for the health professionals developed considering an iterative, User-Centred Design approach, with extensive contributions from a broad set of users including domain experts, resulting in a highly usable and accepted system. Geriatric Helper is currently being tested in Portuguese healthcare units allowing for any clinician to apply the otherwise experts-limited geriatric assessment.

## 1. Introduction

The world’s population is ageing at an increasingly faster pace [1]. Considering the Portuguese context, representative of the reality observed for a large majority of the European Union (EU) countries, the ageing and longevity indices (Table 1) have evolved significantly [2], depicting a strong ageing of the population and an increase in life expectancy, from 72 to 81 years.

This demographic change has a considerable societal impact at different levels and, in recent years, a strong effort has been made to propose methods and technologies to support ageing in place, maintaining an active life, and independent living with a strong focus in areas such as Ambient Assisted Living [3,4] and contributions at different levels [5]. These contributions aim at improving the quality of life of older adults, acting at several dimensions that can positively influence their health status [6]. For instance, to a certain point, a properly medicated, physically and socially active older adult may be able to keep healthier and independent for a longer time [7].

These assistive technologies may provide enough support to keep older adults healthier and away from the healthcare institutions for longer, but several conditions appear, accumulate, or get worst with age, requiring more extensive medical attention. It is this cumulative decline, at different levels, that leads to frailty [8], a state in which any minor health event may have a strong impact on the health status and lead to a longer recovery time (if any). In this regard, the healthcare system needs to provide a proper response in addressing the particularities of older adults [9] with a strong role played by Geriatrics, which is expected to have an increased demand in the years to come, both in healthcare professionals and in treatment facilities.

The coexistence of several complex health states that do not fall into discrete disease categories—geriatric syndromes [10]—is a challenging scenario to assess from a medical perspective, and may lead to frailty, urinary incontinence, falls, delirium and pressure ulcers. These syndromes are associated with significant morbidity and seem to be better predictors of death than the presence or number of specific diseases, but tend to be overlooked in countries that have not developed geriatric medicine as a speciality [11].

### 1.1. Comprehensive Geriatric Assessment

The recognition of the complexity and impact of geriatric syndromes, and the different dimensions influencing the maintenance and restoration of homoeostasis, motivates moving away from single disease diagnosis into a more holistic view of the older adult [8].

Comprehensive geriatric assessment (CGA) [12] is a multidimensional and interdisciplinary evaluation of the elderly. It is composed of mental, functional, nutritional and social areas of assessment, each one containing several related scales to: (a) identify particular conditions; (b) assess and describe, for instance, the level of functional ability; and (c) identify risk levels for certain debilitating factors [13,14]. The main objective of CGA is to reach a precise and full diagnosis and facilitate prevention and follow up [12] of people over 75 years old, or over 65, if in risk situations such as lack of social support, multiple pathologies, chronic disease or institutionalization. Being a multidimensional evaluation [15], it is ideally applied by multidisciplinary teams including physicians, nurses, social services technicians, gerontology doctors and physiotherapists [14]. It should be performed on a regular basis, to better adjust to the decaying health conditions of some patients, and has consistently been associated with lower mortality rates [16,17,18], lower rates of institutionalization [19] and a slower decline of quality of life [20]. Additionally, evidence shows a positive influence of CGA in domains such as cancer treatment, by improving completion rates [21], or vascular surgery, by reducing hospital stays and post-operative complications [22].

After applying the scales judged relevant for each particular case, the health professional may need to prescribe medication. For a diverse set of reasons, such as pharmacokinetic and pharmacodynamic changes due to age, and polypharmacy [23], prescription should be performed with additional care. The Start/Stopp [24] and Beers [25] criteria make up a valuable tool, in this context, since they advise on drugs that should or should not be prescribed to the elderly. However, these criteria encompass large lists that would profit from easier access.

### 1.2. Objectives, Contributions and Overview

The non-existence of a formal medical speciality in Geriatrics, like in Portugal, is a common scenario across many countries [26], making it difficult to widely apply CGA routinely, due to the lack of awareness and training to this kind of evaluation. There are only three medical centres in Portugal where CGA is performed by a multidisciplinary team (nutritionist, medical doctor and pharmacist). For the remaining locations, CGA is applied by a single person, mainly an internal medicine doctor or general practitioner, filing a paper form and computing the result, for each scale, by hand. In this context, our research team was approached by the Geriatric Studies Group of the Portuguese Society for Internal Medicine [27] seeking a collaboration to help them improve the CGA panorama in Portugal. In particular, they would like to:

- Increase awareness and knowledge regarding CGA for those health professionals who deal with elderly patients, on a regular basis, and whose patients might benefit from its application;
- Provide CGA-relevant tools, with a particular emphasis on assessment scales, covering the different dimensions (e.g., functional, cognitive, nutritional) at stake;
- Enable access to clinical criteria regarding medication as a support tool during drug prescription to elderly patients;
- Deliver an approach to CGA that would be easily accessible, in different scenarios, such as emergency care, health centre and nursing homes;

The work described in this article, an extended version of preliminary work presented in [28], addresses these goals by adopting an iterative user-centred approach to design and develop Geriatric Helper, a smartphone application, to be used by the health professionals, supporting the application of CGA. After undergoing several prototyping and evaluation stages, Geriatric Helper is currently being used by several medical doctors in their practice, with good overall feedback.

The remainder of this document starts by analysing the current context for assistive technologies related with geriatric assessment, followed by a description of the methods considered for our work. A characterization of the users and targeted scenarios is then presented, and the main requirements extracted, followed by the description of the iterative design, development and evaluation of Geriatric Helper. Finally, we draw some conclusions and set the directions for future work.

## 2. Mobile Health and Geriatric Patient Assessment

The growing presence of technology in our daily lives, particularly mobile devices such as smartphones and tablets, has opened a range of possibilities for the proposal of assistive technologies for various contexts. Among them, delivering and receiving health information and services through mobile devices—this is called mobile health (mHealth)—has assumed particular relevance [29], boosted by the ubiquity and portability of such devices, by their growing connectivity (e.g., to the internet), and their suitability as hubs for data from multiple sensing technologies (e.g., biometric signals). These mobile solutions are not meant to replace medical doctors, who keep their role of following patients in their use of these assistive technologies [30], but as a complement to traditional medicine; this is particularly important in low-income contexts, where physicians may not be readily available.

mHealth approaches are transversal to a wide set of application domains such as cardiac [31], trauma [32] and pain assessment [33], obesity and diabetes [34], mental health [35,36], medication adherence [37], and chronic diseases [38] and target: (a) healthcare professionals and care givers (e.g., [39]), to support patient assessment and monitoring; (b) patients, from all ages [40], for self-management, and as providers of relevant information for their condition; and (c) for the training of students and professionals, in certain domains (e.g., [41]), and as a way to bring caregivers onboard, by having them adopt guidelines and practises [42,43].

### 2.1. mHealth and Geriatric Assessment

The literature is prolific in proposing mHealth solutions for the elderly targeting, for instance, the assessment of their physical and mental condition based on sensor data (e.g., [44,45]) and the improvement of their condition [46]. However, considering the context of our work, we were particularly interested in tools that could be used by clinicians in the context of comprehensive geriatric assessment considering that, at this point, the most important goal is to ensure that CGA can be performed more easily, is better known, and is systematically applied by more clinicians. To the best of our knowledge, there are only a few mobile solutions focused on assessing the elderly by supporting the evaluation of some of the aspects encompassed by the CGA (see Table 2, for a summary). PT-Measures [47] includes one scale from the mental area and five from the functional area, which are grouped by their respective area, and each scale includes some associated textual information. Indicators of dependence [48] contains 11 scales, from mental, functional and social areas, but they are not grouped into areas. There is detailed information on how to apply each scale, their scoring outcomes and associated bibliography. iGeriatrics [49] was developed by the American Geriatrics Society, does not include information on how to perform CGA, but instead covers a wide range of topics related to older adults, such as vaccination and prevention of falls. It does not allow to apply scales, but provides the Beers criteria. OncoScale [50] includes mental, functional and nutritive scales, grouped into areas along with information about each scale’s bibliography and how to perform it. Plus65 Med [51] is a pocket guide for the Start and Stopp criteria. Criteria can be consulted one by one or a search can be performed for a certain medication, medication class or disease, containing references for each criterion.

The work by Tong and colleagues [52] proposes a tablet application aimed at assessing frailty. Targeting several end-users, such as patients, care givers, and health professionals, it provides a small set of scales for the assessment of the mental, functional and frailty states. Finally, the work by Garm et al. [53], although not a mobile application, is worth mentioning since it proposes a system that, even though it does not fully support all the envisaged dimensions of CGA, explicitly proposes a digital alternative to the paper scales and the application of a reduced subset of CGA built around the assessment of frailty (FI-CGA).

### 2.2. Discussion

Considering our goals, most applications only implement part of the required functionalities, not supporting a structured CGA by covering the required areas and scales. Additionally, those that support a subset of the scales do not allow their consideration as a whole, but as a set of individual tests, which does not transmit the sense of the desired multidimensional evaluation of each patient.

To the best of our knowledge, one important aspect that is also missing in existing applications concerns the formative aspects, an important part of our goals, and recognized as an important requirement to move CGA forward in geriatric care [54]. In fact, this kind of approach has already been considered in other areas [41,42,43,55] with success. However, it is important to note that care should be taken since providing the clinicians with something that only partially supports CGA might, at some point, be taken as defining what a CGA actually is [14], which might limit its potential.

In line with what is observed by Brown and colleagues [39], for dementia care, despite all the advances in mHealth, Geriatrics also has a limited number of apps when we consider the complex needs of its health professionals and caregivers. In a recent work by Theou and colleagues [56], the authors consider it important to examine, in future studies, if CGA could be supported by an application to encourage its use, clarify its value, and enable easier assessment and scoring. The work presented in this article is a first step towards this goal, proposing such an application, Geriatric Helper, currently in use by medical doctors.

## 3. Methods

To tackle the challenges of addressing, as best as possible, the needs and motivations of clinicians when performing CGA, we followed an iterative human-centred design (HCD) approach entailing the involvement of the end-users at all stages of the design and development process [57,58]. This approach makes use of different methods to continuously focus on the needs and requirements of the end-users, encompassing four stages [59]: specification of the context of use; definition of requirements; design and development; and evaluation. These are performed adopting a set of principles [59,60] including the active participation of end-users, in the scope of a multidisciplinary team, and the redesign, as often as required, of the proposed system to meet user needs.

One notable aspect of our approach, which we consider is still aligned with the HCD standard (ISO 9241-210:2010 [61]), is the consideration of audiences other than the end-users to perform the evaluation of usability aspects, particularly during the first development stages. These first evaluations are aimed at solving basic usability issues that could hinder taking the most out of the end-users’ time and feedback and can improve our insight over what might be the causes for any observed issue. We have been following this principle when working with different audiences, for instance: with elderly people [37], for whom a usability issue hindering proper access to application features might discourage their participation and reduce their self-confidence. In addition, this is true for children with autism spectrum disorders [62], for whom existing navigation issues rather than a lack of engagement with system features might hinder their use of a system, which the communication problems, frequently associated with their condition, make harder to discern. In the particular case of this work, initial evaluation stages with non end-users were considered to maximize the outcomes of later evaluation stages with clinicians, focusing the discussion on more relevant aspects. Note however, that non end-users are never considered to set or refine requirements.

### 3.1. Requirements Definition

In the adopted methodology, Personas [63,64] play a central role by supporting the definition and characterization of the targeted users. Personas are fictional characters, with name, occupation, age, gender, socio-economical status, interests, stories and motivations, and are used to represent the end-users and their relation with the problem and context at hand [65,66]. They are, overall, a way to humanize the users and favour empathy with their goals and needs.

To obtain the requirements, we followed the overall methodology proposed by Cooper et al. [63], summarized by the following steps:

- **Personas:** Identification of main characteristics of the targeted users, such as age, activities, motivations and skills and their consideration to create the basic features of the Personas and define their goals. Definition of the different types of Personas (primary, secondary, served);
- **Vision Statement:** Production of a brief statement for the problem and vision, to provide a focus point for the following steps;
- **Brainstorming:** Idea gathering from a set of people including different domains of expertise and the end-users, in the form of a brainstorming session, by openly discussing all aspects regarding the problems, users, and application domain;
- **Context Scenarios:** Based on the outcomes of the brainstorming session, and vision, definition of expectations for the Personas and a set of scenarios illustrating how the intended system will address different dimensions of end-user expectations. Scenarios depict what the system will do, before settling on how it will do it;
- **Requirements:** The devised scenarios and Personas set the context from which requirements are extracted.

After this initial requirement definition stage, each iteration of the methodology, considering the outcomes of the evaluation, dictates the refinement or addition of requirements.

### 3.2. Prototype

The prototypes are an important asset to mediate the contact between the design and development team and the end users [67]. The short development stages enable the addition of a selected set of novel features, based on priorities, and the resolution of issues providing an increasingly tangible base for discussion without consuming too many resources. This is important in all application domains and audiences, but assumes a particular relevance when developing for users who need to integrate a particular system into an existing workflow, such as medical doctors [68], enabling the detection of unpredicted barriers at an early stage.

### 3.3. Evaluation

Evaluation is performed at the end of each development cycle and designed taking into consideration different purposes and choosing the participants accordingly. The methodology considered for evaluation [69] proposes three different evaluation stages: conceptual validation, prototype test and pilot test. The conceptual validation determines the viability of the application interface and functions. Prototype tests include the participation of users, interacting with the application, and typically involve an observer that, as a complement to other assessment tools, collects data regarding user difficulties and suggestions. These tests are aimed at obtaining feedback to improve and expand the application. Finally, pilot tests evaluate how the proposed application impacts the end-users. How we move from one evaluation stage to the other mostly depends on the outcomes of each iteration. Moving into a pilot test, for instance, entails having a reasonable set of useful features already validated by previous evaluations to effectively enable its real use.

## 4. Users, Scenarios and Requirements

The first stage consisted of a brainstorming session involving a multidisciplinary team composed of a mobile computing expert with experience in mHealth, an interaction designer, a software developer, and a medical doctor from the Geriatric Studies Group (in Portuguese: Núcleo de Estudos de Geriatria—GERMI) [27], of the Portuguese Society of Internal Medicine. The outcomes of this session guided the creation of first versions of the Personas complemented, later on, with additional information collected from the medical doctor, the discussion and approval of context scenarios, and subsequent definition of requirements.

### 4.1. Personas

The design and development of Geriatric Helper was supported on four Personas deemed relevant for a proper characterization of the CGA context [63]: a primary Persona, Francisco, an Internal Medicine Physician (Box 1); a secondary Persona, Alberto, a general practitioner (Box 2); and two served Personas, Lurdes (Box 3) and Mário (Box 4), geriatric patients.

Box 1 **Primary Persona: Francisco, an Internal Medicine Physician.**

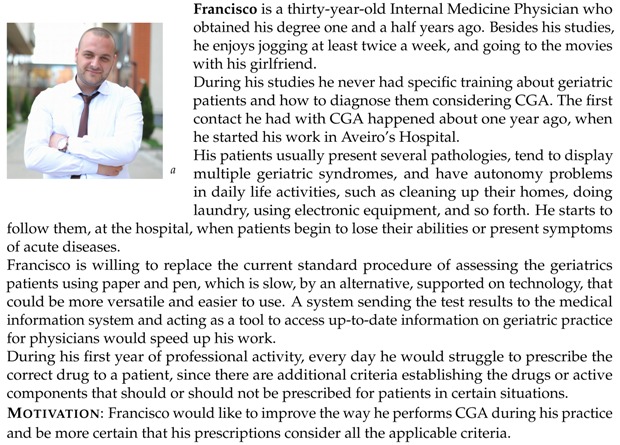

*a* Image adapted from https://pxhere.com/en/photo/625568.

Box 2 **Secondary Persona: Alberto, a General Practitioner.**

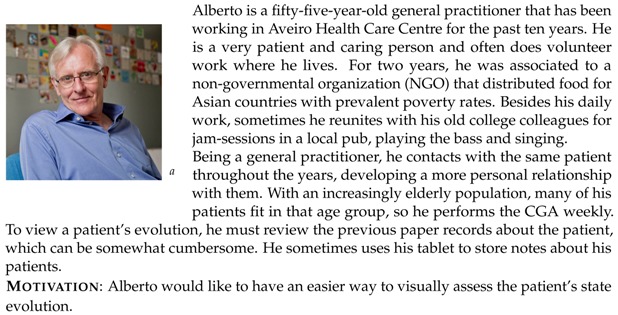

*a* Image adapted from https://pxhere.com/en/photo/84857.

Box 3 **Served Persona, Lurdes, a geriatric patient attended at the Hospital.**

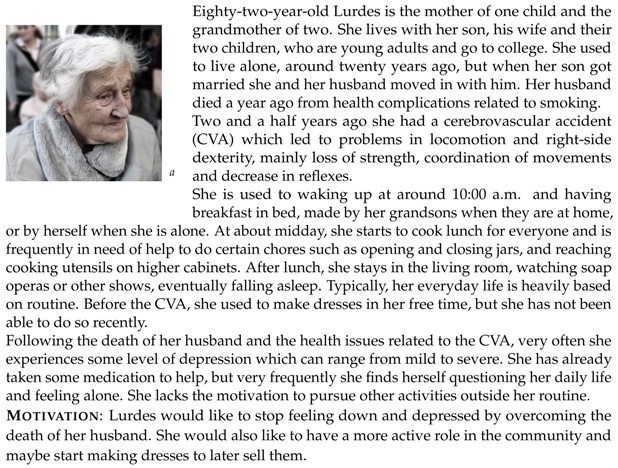

*a* Image adapted from https://pxhere.com/en/photo/1132190.

Box 4 **Served Persona, Mário, a geriatric patient attended at the Health Center.**

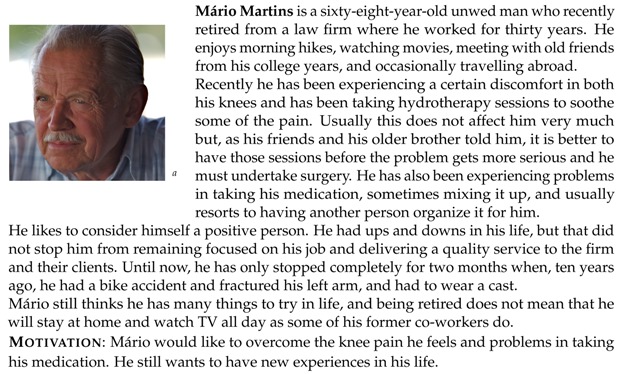

*a* Image adapted from https://www.flickr.com/photos/liberato/200858281.

Even though our aim at this stage was not to consider any feature for patients, they have been included as served Personas to enable a more complete understanding of the application scope since they benefit from its successful use. In the case of the general practitioner, Alberto, a distinct profile from the primary Persona, he is considered as a secondary Persona since, in a first approach, his needs could mostly be satisfied by the design options considered for the primary Persona. However, during the iterative process, we became aware of a different clinician profile, with less technical skills, who would profit from an application more adaptable to different levels of experience and supplementary guiding materials, leading to the inclusion of this new Persona.

### 4.2. Scenarios

The context scenarios depict how the proposed system integrates with the end-users’ activities to enhance how they perform them towards fulfilling their motivations. For understanding the different ways in which Geriatric Helper would be used, the initial brainstorming session resulted in several context scenarios that were then reviewed by clinicians to ensure that they made sense and reflected an integration of Geriatric Helper that was plausible. This was a first step (the second being the pilot test) towards ensuring that the proposed approach was compatible with their workflows. Here, for the sake of brevity, we only present three illustrative scenarios. The first scenario depicts Francisco opening the application and getting acquainted with the different features.

**Francisco performs a CGA assessment**—Francisco opens the application and sees a welcome message which informs him about the key features of the application. He is informed that only the standard features are activated, and additional functionalities are available for activation from the menu. He explores the information regarding the application of CGA and reviews some of the scales that he usually applies during his practice. He experiments with filling a few and checks the computed results.

In the second scenario, Francisco is assessing Lurdes’ current health status. He already knows her from previous consultations and wants to check how her situation evolved.

**Assessing Lurdes’ Condition**—Before starting the assessment, Francisco and Lourdes have a brief chat about how she felt in the last month. The physician thinks that Lurdes has been sadder recently since this month marks the anniversary of her husband’s death. The last time they met, he applied the full CGA, to her. Today, he applies the Yesavage test for tracking depression states in elderly patients. He checks her temporal evolution for the test and sees that the results have worsened since the last appointment, pointing to a state of mild depression. He opts to prescribe some drugs.

Finally, Francisco needs to prescribe medication and, considering Lourdes’ condition, uses Geriatric Helper to access clinical criteria.

**Prescribing medication to Lurdes**—The physician needs to be careful since Lourdes already takes medication for other health issues and the drug he is going to prescribe must not interfere, causing new health complications. Since he needs to consult medication-related criteria, which is not visible by default, he heads to the settings menu. He browses a list of the modules that can be activated and deactivated and proceeds to activate the Clinical Criteria module. Francisco consults the app and searches for the name of the medicine he has in mind. The app tells him that medicine should be avoided for a health issue Lourdes already has. Francisco then proceeds by using the Start criteria that inform which drugs are best for certain conditions.

The underlined excerpts, in the presented scenarios, hint at the actions that were relevant for identifying the requirements.

### 4.3. Requirements

Building upon the scenarios jointly-elaborated with the domain experts, an initial set of requirements was defined, to guide the development of the first prototype (see Table 3). These first requirements focused the creation and application of the assessment scales. Subsequent evaluation revealed, for instance, that additional tutorial contents were needed to help new users to succeed in the application of scales (the main topic of the second prototype development). A practical feature, not foreseen at early stages, was the ability to export the applied scales into a portable document that users could print or save for future reference. It is also interesting to note that a broader group of domain users, at a later stage of development, identified that it would be helpful for the adoption of Geriatric Helper if it was possible to set a basic level of usage (just the scales), for less experienced users, while advanced users could still enable other features, such as the patient records management.

## 5. Geriatric Helper

Four prototypes were developed, starting from a subset of the initial requirements, and the outcomes of each iteration were the subject of user assessment, to detect problems and refine the requirements. After the fourth prototype, the application was deemed adequate to undergo a pilot test. Table 4 provides a summary of the main aspects of each iteration including the main goals, considered evaluation methods and participants, duration of the iteration (distinguishing between development and evaluation times) and the devices involved in evaluating the prototype.

In an initial stage, we considered that the most important goal was to rapidly propose a proof-of-concept that could be validated by the clinicians and, therefore, opted for the development of a single platform (Android). Later, considering the number of requests to support other platforms, an iOS version was also deployed.

In what follows, a brief description of the main aspects of each iteration, including the outcomes of each evaluation stage, is presented.

### 5.1. First Iteration

**Figure 1:**
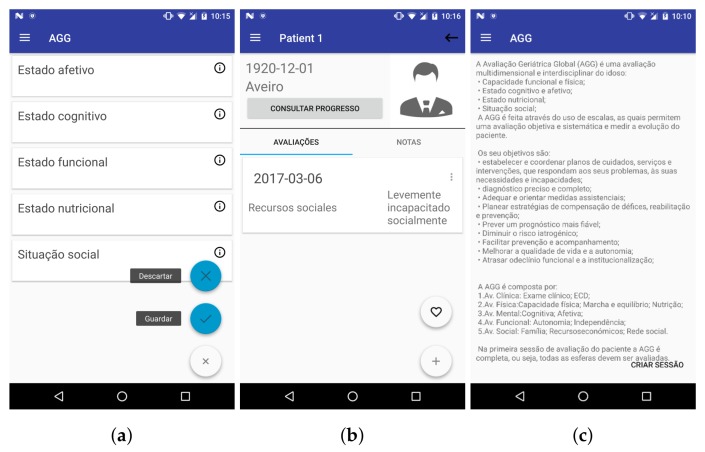
Several screen captures of Geriatric Helper’s first prototype: (**a**) menu depicting the different evaluation areas (affective, cognitive, functional, nutritional and social); (**b**) current status for ongoing session, with the social status assessment already performed (outcome: mild social incapacity); and (**c**) overall description of CGA purpose and components, deemed an issue to correct, during the heuristic evaluation, due to the amount and size of the text.

The first prototype was based on the initial system requirements and it allowed performing public and private CGA sessions, consulting medical criteria, and managing patients’ profiles, along with their respective sessions (Figure 1).

The iterative method followed considers that the evaluation type should be adapted to the current stage of development. The objective for the first prototype’s evaluation was conceptual validation, mainly assessing issues concerning the overall usability of the application, with the context specific features assessed at later stages. Accordingly, we performed a heuristic evaluation and, considering the purpose of this development stage, the users who participated were not yet domain experts, but five students with Computer Science degrees (at the University of Aveiro) with previous experience with heuristic evaluation. For the list of heuristics to consider, we included Nielsen’s heuristics [70], the Competency concept from Health-ITUEM [71,72], “Pleasurable and respectful interaction” and “Privacy heuristics” from the heuristics proposed for mobile interfaces [73]. The consideration of these heuristics, besides Nielsen’s, aimed to cover other aspects we deemed relevant in this context without rendering the evaluation very time consuming as it would result from the inclusion of the full three heuristic sets.

Most of the usability problems found were not severe to the point of making it impossible to use the application and its main features. The heuristic that was mostly infringed was “Aesthetic and minimalist design”, in both versions of the application (smartphone and tablet), with reasons such as “There is too much text” (see Figure 1c) or “Text is too small” .

### 5.2. Second Iteration

**Figure 2:**
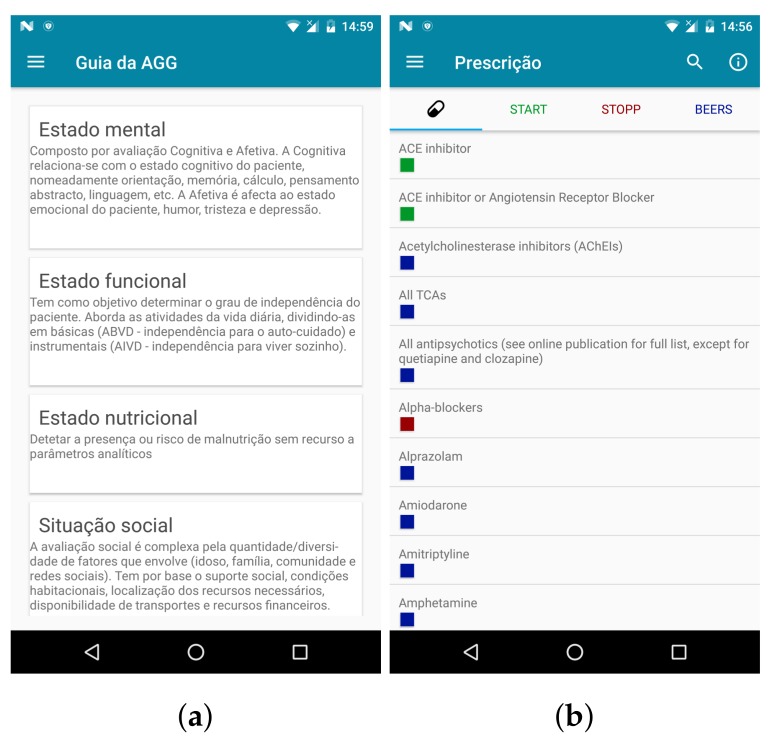
Screens for the second prototype of Geriatric Helper: (**a**) guides available regarding the different assessment dimensions (visible: cognitive, functional, nutritional and social); (**b**) clinical criteria to observe when prescribing for elderly patients.

The second prototype added the possibility of user registration through the application, contained improvements to patients and sessions management, allowed adding textual notes to a patient, allowed the application to run on more devices, sessions appeared in a dedicated menu entry, it was easier to quickly check which type of requirements were associated to a drug, and tests information could be consulted as a pocket guide (Figure 2).

The evaluation of the second prototype aimed to detect additional usability issues that become apparent when the application is being used to attain concrete goals. A think aloud protocol [74] was adopted to gather usability data, with participants thinking aloud while performing a set of 15 tasks. These included goals such as “Check patient’s progress relative to Clock Drawing scale” or “Consult Start criteria associated with Metformin”. The participants in this evaluation were 11 Computer Science postgraduate students with knowledge of Human Computer Interaction (HCI) and/or mobile applications development.

Besides measuring the time taken to complete a task and the success rate, we deemed it necessary to obtain additional information regarding the usability and easiness while handling the application. To this end, the literature proposes several post-study questionnaires, including the Software Usability Measurement Inventory (SUMI) [75], Post-Study System Usability Questionnaire (PSSUQ) [76], and the System Usability Scale (SUS) [77]. The first one requires purchasing a license so it was dismissed from the start. PSSUQ should be used carefully as it is susceptible to the “acquiesce bias” (people are more likely to agree with a statement than to disagree with it), since all questions in the PSSUQ are positively worded. Therefore, our choice was the SUS avoiding the other methods’ drawbacks. The original version of the SUS is in English [77] but, when applying it to a Portuguese group of participants, some of them may not be as capable of understanding the language as others. Therefore, we used an existing validated version of this scale for European Portuguese [78].

**Figure 3:**
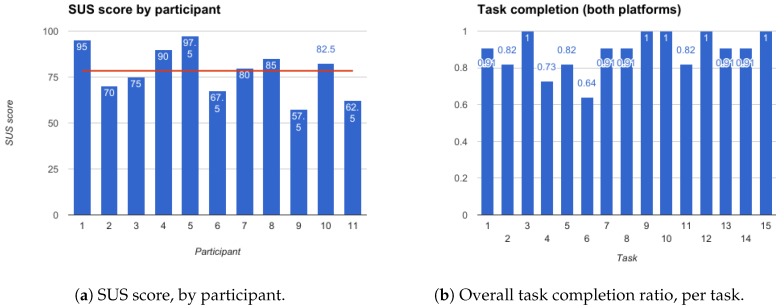
Results obtained for the evaluation of the second prototype of Geriatric Helper: (**a**) the System Usability Scale Scores (SUS); and (**b**) task completion ratio.

Overall, the participants were able to complete the tasks (see Figure 3a), with an average success rate of 89%, although a few required more time to get acquainted with the application and exceeded the empirically estimated time for task completion. Regarding SUS results (Figure 3b), the average was 78.4, the lowest 57.5 and the highest 97.5. Considering that a SUS score of 68 is indicative of a good usability and satisfaction level [79,80,81], the obtained score positions the results above average and, thus, Geriatric Helper could already be considered as providing a good usability, at this stage.

After the evaluation, and after solving some of the usability problems reported during the think aloud, a brainstorming session was conducted with a clinician, presenting the current state of the application and collecting suggestions that further informed the creation of the third prototype.

### 5.3. Third Iteration

In this iteration, we made several corrections to improve contextualization, such as displaying a summary of each CGA area when conducting a CGA session, or showing the patient name, at the top, during an ongoing assessment. Additionally, error prevention was also improved, to minimize data loss, introducing warnings when trying to leave a scale without having answered every question, and adding feedback to actions that were lacking it. The added functionalities were the possibility to generate a PDF for a CGA session, which later could be archived on the patient’s medical file, and the ability to keep a list of drugs prescribed for each patient.

Since the most prominent usability issues had already been solved, in the previous evaluation stages, the evaluation of this prototype was performed by clinicians using a less structured approach, based on an open analysis and discussion among peers. The clinician previously involved in setting the context and requirements presented the application to the physicians from the Geriatric Studies Group (GERMI), composed of eight physicians of different ages and experience levels, and they were free to use the app, install it on their devices, ask questions and comment on its features.

Overall, this evaluation resulted in two recommendations, mostly related with the capability to adapt to different levels of experience and proficiency of the end-users: (1) start the application with a basic set of features and allow more experienced users to activate additional, more advanced modules; (2) the inclusion of additional help contents, providing a guided tour to the basic features available.

Analysing the outcomes of this evaluation, we concluded that some of the participating physicians were less proficient with smartphone technologies, so they presented a sufficiently different profile to justify the inclusion of an additional Persona, the general practitioner Alberto.

### 5.4. Fourth Iteration

**Figure 4:**
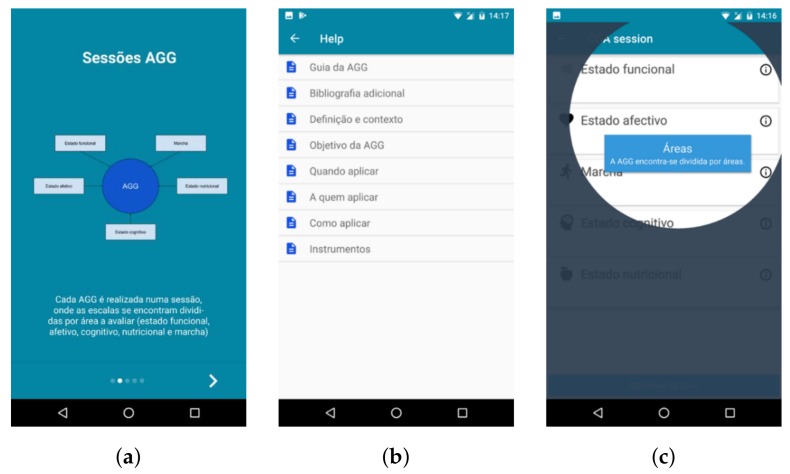
Screens for the (**a**) tutorial home screen, providing a short explanation about CGA, (**b**) help menu, 0 and (**c**) guided tour features added to the fourth prototype of Geriatric Helper.

Most of the changes made in this prototype were aimed at helping users with different experiences and goals use the application (Figure 4). Some users prefer a simpler application, with less features (Alberto), while others may want to have access to all available features (Francisco). The progressive activation of advanced functionalities makes the initial state of the application less overloaded with information, which will allow for a more intuitive first use and a more sustained learning curve.

The key modules include creating a CGA session to assess a patient and consult the CGA guide while outside of an appointment (Figure 4b). The extra modules, as defined by the physicians, were Prescription (browse clinical criteria), Personal Area (including patients’ management) and Other Scales (certain scales can be activated and deactivated).

To make the application more intuitive and easy to use, several “tutorial” functionalities were included, such as videos explaining how the application works and creating an interactive guide accompanying the user step-by-step when conducting the first CGA session (Figure 4c).

**Figure 5:**
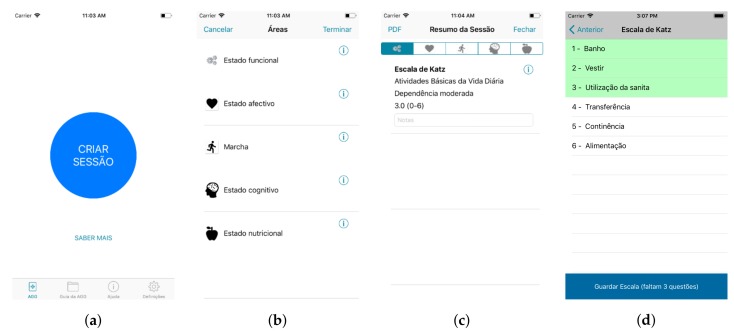
Geriatric Helper for iOS: (**a**) start screen; (**b**) menu of the different dimensions that can be assessed; (**c**) reviewing the outcomes of the ongoing assessment session; (**d**) items to be assessed in a scale.

During the evaluation of the third prototype, several physicians expressed their interest in the features provided by Geriatric Helper in its current status, deeming them already interesting and relevant for their daily practice. This feedback regarding the perceived usefulness of Geriatric Helper and the existence of several physicians with iOS smartphones, led to the consideration of an iOS version (Figure 5), having as base the approach and current status of the Android version.

#### 5.4.1. User Interface

The user interface, at iteration four, is partially illustrated in Figure 5 for the iOS implementation. The welcome screen will lead the user to the creation of a new session, providing a large, prominent button (Figure 5a). For performing the Comprehensive Geriatric Assessment, different assessment dimensions or areas can be considered. They are consistently identified in the application with an icon (Figure 5b). In this list, the user can also easily obtain a description of each dimension, by selecting the information icon. When the user picks an area (e.g., functional state, the first), a list of scales concerning this area are listed. If they have been previously assessed, a summary of the outcomes is shown. Figure 5c shows the results for the Katz scale and informs that a moderate dependency in daily life activities occurs. To apply the different scales included in the protocol, the user selects and “opens” the different scales and fills each question (Figure 5d). The application informs, in green, the questions already answered, making it clear when the user is still expected to complete the assessment with more data. Key actions, such as closing the dialogue and returning to the previous view, are available from the top bar and integrated in the native navigation. This user interface has been presented to the domain experts and interactively evolved as detailed in the previous sections.

#### 5.4.2. Pilot Test

Considering that the current prototype already serves a refined set of the initial requirements, it was considered appropriate to undergo a pilot test. The objectives were to use the application in a real context, with physicians, to retrieve their feedback, serving a more effective evaluation of the adequateness and relevance of Geriatric Helper for CGA. The pilot test is a major step since it implies the use of the application in a real context and is an important source of information to evaluate how the proposed application actually integrates with the physicians’ workflow, a rather important characteristic for its acceptance [68].

Not all medical doctors perform CGA and, therefore, to cover a wide population of users, the pilot test would need to run at different healthcare facilities, geographically distributed across Portugal. Therefore, it would not be feasible to have full control over who is performing it and formally collect feedback. In this regard, we adopted a mixed approach that enabled some formal feedback side-by-side with a broader use by clinicians connected with GERMI, enrolled directly by other members or motivated by our presentation of Geriatric Helper in their annual meeting. While for the first, we would obtain direct feedback, the remaining feedback would reach us through GERMI.

The more structured pilot test was performed with two clinicians, members of GERMI, with at least one year of experience in applying CGA, and was divided into three stages: (a) initial task-driven think aloud session; (b) pilot stage; and (c) a SUS questionnaire. First, we considered that an initial session for presenting Geriatric Helper to each physician would be important to highlight the main features available. However, instead of just making a presentation, this session was designed to obtain feedback on first impressions and difficulties by structuring it as a goal driven think-aloud session: participants performed tasks and commented on their perceived difficulty. This way, they got acquainted with the main features and we got additional feedback on potential problems.

After this session, the clinicians started using the application for a few weeks and were provided with a form where they could annotate any comments or report issues. Finally, they filled a satisfaction questionnaire (SUS), which conveyed quantitative results to be further analysed.

During the think-aloud stage, participants found goals to be easily achieved, with easiness levels ranging between 4.5 and 5, and times taken to achieve goals were as anticipated. As expected, every goal was successfully completed, since no tester was unable to complete the tasks and no tester exceeded the indicative time limit.

The comments and suggestions made by testers during this part of the pilot stage concerned details that are only perceived after an extensive use of the application and which are easy to address in the next version of Geriatric Helper. Most of them were suggestions to improve the system’s functionality and the most significant issue reported was the application being prone to crashes when completing the Mini Mental State Examination scale. Positive comments regarding the app’s intuitiveness and the fact that it envisages everything a clinician needs when conducting the CGA are a good indicator that this technological solution to the CGA is well implemented and valuable to physicians. Analysing the SUS results from the two users (90 and 97.5), the obtained score is very good.

At this time, the application has been downloaded by 103 users (44 Android and 59 iOS) and, considering the informal feedback that has been provided, we anticipate an overall positive acceptance by clinicians.

## 6. Conclusions and Future Work

We present a new method to perform a CGA based on a mobile application, shifting from a paper-based approach to one supported on a mobile device. The application was developed considering a user-centred approach and its functionalities were designed considering the Portuguese reality, with the active participation of domain professionals. It is an improvement to the current practice, enabling easier calculation of scale scores and browsing of clinical recommendations. Geriatric Helper has been tested throughout the development process and it is currently undergoing a pilot test, being used by medical doctors during their practice with elderly patients.

The options considered for evaluation of the different prototypes have enabled making the most out of the clinicians time, by solving more evident usability issues before they tested the application. Additionally, opting for a less structured evaluation session for prototype 3, with an open discussion among clinicians, provided valuable information on how to adapt Geriatric Helper to a broader range of clinicians.

Geriatric Helper is already a valuable tool to support performing CGA and, in line with what Theou et al. [56] suggest, it would be important to conduct a long-term evaluation of its use and the impacts of its application in patients’ prognosis.

At its current development stage, the proposed application can also work as grounds for additional evolutions, not only to further enhance how it supports CGA, for instance improving how it enables patient management by integration with wider patient registries (along with the authorizations and additional security it requires [82]), but also adding to how the clinician can obtain relevant patient information. In this regard, the progressive inclusion of a self-assessment [83] component and sensing technology [84] might bring additional objectivity and information to the clinical assessment.

Although this also holds true for medical doctors, the consideration of self-assessment components, to be used by elderly users, will require an increased emphasis on the interaction design, particularly addressing accessibility for audiences with different abilities. In this regard, further evolving the elderly Personas proposed in this work along with an adaptive multimodal interactive interaction design should provide a suitable approach [85]. 

## Figures and Tables

**Table 1:** Evolution of the ageing and longevity indices, in Portugal, comparing the values for 1980 and 2013. The European Union (EU) values are provided as a reference for the European panorama. Source: [2].

Index	Portugal		EU
	1980	2013	2013
ageing (%)	43.8	133.5	117.5
(≥ 75 yo/ < 15 yo) × 100			
longevity (%)	14.4	27.7	27.7
(≥ 80 yo/ > 65 yo) × 100			

**Table 2:** List of applications providing features related with geriatric assessment and their characteristics deemed relevant for the overall context of Comprehensive Geriatric Assessment.

Application	Areas Covered by Scales	Info About Scales	Patient Follow-Up	Clinical Criteria	Lang.	Platform	Ref
PT-Measures	mental, functional; grouped by area	+	-	-	PT	mobile	[47]
Indicators of dependence	mental, functional and social; not grouped by area	++	-	-	PT	mobile	[48]
iGeriatrics	-	-	-	Beers	EN	mobile	[49]
OncoScale	mental, functional and nutritional; grouped by area	++	-	-	FR	mobile	[50]
Plus65	-	-	-	Start/Stopp	EN	mobile	[51]
Computerized Frailty Assessment	mental, functional, frailty	-	+	-	EN	mobile	[52]
FI-CGA	mental, functional, nutritional and social	-	+	-	EN	desktop	[53]

**Table 3:** Requirements considered along the iterative development of Geriatric Helper, for each iteration (prototype).

Prot.	Subset of the Original Requirements List Implemented
1	1. Create new assessment sessions
	2. Apply the CGA scales to assess a patient’s condition
	3. Persistence of the current CGA session to enable resuming it
	4. Create a local, secure patient record
	5. Browse saved patients
	6. Allow CGA sessions to be saved into a patients’ records
	7. Track a patient’s evolution (key parameters along time)
	8. Access medication guidelines from the app (for the elderly)
2	9. Act as a guide on how to conduct a CGA evaluation (tutorial approach; facilitate on-boarding of new adopters)
	10. User registration from inside the application
3	11. Export a CGA session (assessment results) into a PDF document
	12. Share results (send PDF to other apps)
4	13. Prescribe drugs to a patient, cross-checking against guidelines
	14. Save drugs prescribed to a patient
	15. Strong focus on "help" contents, such as explaining how to use the app for the first time by means of a guided tour of the procedure to apply one of the CGA scales
	16. Allow to configure different feature sets in the application, in particular, enable a basic level, without patient records management and prescription criteria.
	17. Support the iOS mobile platform

**Table 4:** The iterative user-centred design and development of Geriatric Helper comprised four prototypes. The evaluation method was adapted to the focus and development stage and the brainstorming sessions always included clinicians.

Iter.	Duration		Development Goal	Evaluation	Participants	Devices
	10 h		Brainstorming sessions to define users, scenarios and requirements			
1	devel.: eval.:	3.5 mo 0.5 mo	Initial version of the application, minimal viable product	Heuristic Evaluation	5 postgraduate Computer Science students	A, C, D
2	devel.: eval.:	2 mo 1 mo	Correct issues detected in the first prototype, add more functionalities (like improvements to patients and sessions management)	Task-based with Think aloud + SUS	11 postgraduate Computer Science students (aged 23–42)	A, C, D
	2 h		Brainstorming session with clinician to discuss the current version			
3	devel.: eval.:	1.5 mo 0.5 mo	Further develop the concept of patients and session management, explore multiple workflows for the same action, improve the CGA guide; assessment by geriatric physicians, to reach a functional validation of the app	Assessment with end-users/Open discussion and testing	1 clinician for preparing evaluation +8 clinicians with CGA experience	B, C, D
4	devel.: eval.:	2 mo 3 mo	Meet particular details identified by the end-users in the third prototype’s assessment; deployment of the iOS version	Initial task-based think aloud + SUS/Pilot test with end-users	2 clinicians in structured approach +31 ongoing tests	B, D, E, F

**Table:** 

List of devices				
**Id**	**Platform**	**Version**	**Class**	**Model**
A	Android	7	smartphone	Huawei Nexus 6P
B	Android	8	smartphone	Huawei Nexus 6P
C	Android	6	smartphone	Motorola Moto G
D	Android	5	tablet	Asus Zenpad 8S
E	iOS	11	smartphone	IPhone 6
F	Android	multiple	smartphone	Several models (owned by the health professionals), corresponding to 31 devices.
